# Daily touchscreen use in infants and toddlers is associated with reduced sleep and delayed sleep onset

**DOI:** 10.1038/srep46104

**Published:** 2017-04-13

**Authors:** Celeste H. M. Cheung, Rachael Bedford, Irati R. Saez De Urabain, Annette Karmiloff-Smith, Tim J. Smith

**Affiliations:** 1 Centre for Brain and Cognitive Development, Birkbeck, University of London, London, UK; 2 Biostatistics Department, Institute of Psychiatry, Psychology & Neuroscience, King’s College London, London, UK; 3 Psychological Sciences, Birkbeck, University of London, London, UK

## Abstract

Traditional screen time (e.g. TV and videogaming) has been linked to sleep problems and poorer developmental outcomes in children. With the advent of portable touchscreen devices, this association may be extending down in age to disrupt the sleep of infants and toddlers, an age when sleep is essential for cognitive development. However, this association has not been demonstrated empirically. This study aims to examine whether frequency of touchscreen use is associated with sleep in infants and toddlers between 6 and 36 months of age. An online survey was administered to 715 parents reporting on child media use (daily exposure to TV and use of touchscreens), sleep patterns (night-time and daytime sleep duration, sleep onset - time to fall asleep, and frequencies of night awakenings). Structural equation models controlling for age, sex, TV exposure and maternal education indicated a significant association between touchscreen use and night-time sleep, daytime sleep and sleep onset. No significant effect was observed for the number of night awakenings. To our knowledge, this is the first report linking the use of touchscreen with sleep problems in infants and toddlers. Future longitudinal studies are needed to clarify the direction of effects and the mechanisms underlying these associations using detailed sleep tracking.

Sleep is the dominant activity of an infant and plays an important role in neurodevelopment and synaptic plasticity^1^,^2^,^3^. Both the brain and sleep patterns undergo parallel and substantial developmental change during the first few years of life. Given that neural plasticity is at its greatest during infancy and toddlerhood^4^, sleep is likely to have the most impact on the brain and on cognition during this critical period of early development. Yet, around 20–30% of young children experience problems with sleep^5^. One known environmental contributor to poor sleep is the heavy use of screen media, such as TV and videogaming (see review 6,7). In recent years, family ownership of touch screen devices has risen rapidly (from 7% in 2011 to 71% in 2014)^8^. Reports from 2016 indicated that 86% of UK family homes have access to the Internet, with access mainly via portable media devices^9^. For infants and toddlers, touchscreen devices offer an intuitive and attractive source of stimulation^10^, and their portability allows for a wide range of use across multiple settings^11^. Yet, the widespread use in this age group has raised serious concerns for parents, educators and policy makers, as the potential impact of touchscreen use on toddler development, such as sleep, remains unknown. In this study, we use data from a large UK survey (see ref. 12 for details) to investigate the relationship between touchscreen use and sleep in infants and toddlers between 6 and 36 months of age.

Problematic sleep in children is not uncommon. A recent longitudinal study mapped the sleep trajectories in around 3000 children from birth to 7 years and found that 60% of the children have atypical sleeping patterns: the majority of whom were initially short sleepers (45%), others were either persistent short sleepers (12%) or poor sleepers (3%). Compared to the typical sleepers, all three groups showed some degree of impaired physical, emotional and social functioning^13^. The results suggest that, even though some infants with atypical sleep patterns early on eventually develop typical patterns of sleep by 6 or 7 years of age, reduced sleep duration in the first two years of life may have long-term consequences on later developmental outcomes. These findings are mirrored by several follow-up studies in children and adolescents, showing significant associations between sleep difficulties or irregular bedtime and later problems with mental and physical health and lower cognitive and academic performance^13^,^14^,^15^,^16^,^17^. As such, specific guidelines have recommended screens to be kept out of a child’s bedroom specifically because of the potential impact they may have on sleep^9^,^18^. To date, research into the long-term impact of poor sleep during early development remains limited. Yet, findings so far converge, linking shorter sleep duration to negative developmental outcomes.

Media use has frequently been linked with inadequate sleep in children and adolescents (see 6,7 for review). The majority of studies (~90%) show a consistent pattern linking increased screen time with shorter total sleep time and delayed bedtime. This association was observed across various types of media including TV, computer and mobile phone devices. Research on touchscreen media and sleep in children is by comparison, more limited. A recent meta-analytic review identified 20 studies in children and adolescents aged between 6 and 19, and found strong and consistent evidence for detrimental effects of portable touchscreen devices on sleep quality and quantity^19^. Specifically, individuals who are exposed to or have access to a portable media device at bedtime have significantly reduced night-time sleep and increased poor quality of sleep (defined as difficulties in sleep initiation and maintenance). To date, only a handful of studies have explored the impact of screen media on sleep in preschool infants and toddlers – all of which included only TV as media type^20^,^21^,^22^,^23^,^24^. All studies used parent questionnaires, and have reported a significant effect of screen time on sleep: increased amount of TV viewing was associated with parent-reported sleep problems^24^, shorter night-time sleep duration^20^,^22^, reduced quality of sleep^23^, and irregular naptime and bedtime schedules^21^, adjusting for known confounds including socioeconomic status (SES). Other maternal or child characteristics were also included in some studies, including maternal age, education, pregnancy BMI; child’s ethnicity and gender^20^,^22^. Portable touchscreen devices may exacerbate the problem by allowing small children to increase screen time throughout the day and carry the screen into their sleeping space. Yet, so far no studies have examined the impact of touchscreen use on infant and toddler sleep.

In our UK-based survey on 715 families, we reported that 75% of toddlers between 6 months and 3 years of age use a touchscreen on a daily basis^12^. This figure is similar to another study from the UK^25^ and to reports from other countries in both high^10^ and low SES communities^26^. In our sample^12^ we found that the prevalence of daily use increases substantially with age, from 51% in 6- to 11-month-old infants to 92.05% by 25–36 months. Even among the 25% of children who did not use a touchscreen daily, only 42% reported no prior use. Among users, daily usage increased with age from 8.53 minutes a day (6–11 months) to 45 minutes a day (26–36 months). Given the evidence that 1) media use is linked to poor sleep in older children and adults, 2) touchscreen use in infants/toddlers is highly prevalent, and 3) sleep plays a prominent role in early cognitive and brain development, it is critical to investigate whether touchscreen use is associated with sleep problems early in development.

Using a large online survey, this study aims to investigate whether the frequency of daily touchscreen use is associated with sleep in infants and toddlers between 6 and 36 months. Parents were asked to report on the average duration of their child’s daytime and night-time sleep, the time taken for their child to fall asleep, as well as the frequency of night awakenings, to obtain a comprehensive account of infant/toddler sleep patterns.

## Results

As we have reported in our previous study^12^, the average touchscreen usage in this sample is 24.44 minutes (Table 1). Descriptive statistics for the sleep variables split by age quartiles are also presented in Table 1. Modest but significant correlations are observed amongst the sleep variables, with the exception of daytime sleep and sleep onset, which were not significantly correlated (Table 2).

**Figure 1:**
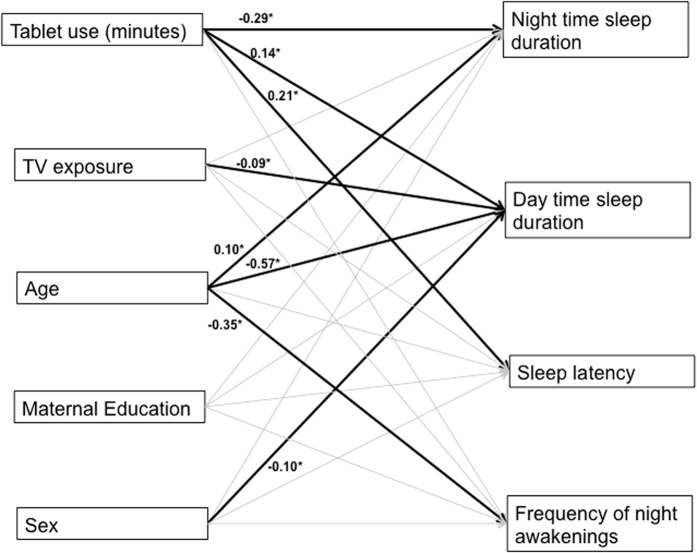
Path diagram showing the association between tablet use and sleep, controlling for TV exposure, age, maternal education and sex (0 = male, 1 = female). Solid black arrows with standardized coefficients represent significant pathways, grey arrows show non-significant pathways. Correlations between the predictor variables and between the outcome variables were included in the model but are not shown in the figure for simplicity.

To examine the relationship between touchscreen use and sleep, a saturated path analysis model was run (see Fig. 1), with the sleep variables (duration of sleep at night, duration of sleep during the day, sleep onset, and frequency of night awakenings) as correlated dependent outcomes, and tablet use, as well as several covariates (average duration of daily TV exposure, age, mother’s education, and sex) as predictors. There was a significant association between touchscreen use and duration of sleep at night (beta = −0.291, SE = 0.062, p < 0.001), duration of sleep during the day (beta = 0.139, SE = 0.068, p = 0.042), and sleep onset (beta = 0.213, SE = 0.051, p < 0.001), with increased touchscreen use associated with decreased night-time sleep, increased daytime sleep and a longer sleep onset. There was no significant association between touchscreen usage and frequency of night awakenings (beta = 0.068, SE = 0.044, p = 0.122).

Results also showed a significant association between average duration of daily TV exposure and duration of daytime sleep, independent of frequency of tablet use (beta = −0.093, SE = 0.040, p = 0.020), with increased TV exposure associated with less sleep in the day. No other associations between TV exposure and the sleep variables were found (p values > 0.134). Sex was also only associated with sleep during the day (beta = −0.103, SE = 0.036, p = 0.004) with boys sleeping more than girls. The effect of age was significant across all sleep variables (except time to put to sleep; p = 0.228), with older children showing increased duration of sleep at night (beta = 0.099, SE = 0.045, p = 0.028), decreased duration of sleep during the day (beta = −0.574, SE = 0.031, p < 0.001), and less night awakenings (beta = −0.349, SE = 0.038, p < 0.001). No significant effects of maternal education were found (p values > 0.107).

To test whether touchscreen use was significantly associated with the total amount of sleep (i.e., day time + night time sleep) we re-ran the model replacing the outcome variables sleep at night and sleep during the day with their sum ‘total sleep’. The other associations specified in the model remained the same. Results showed that increased touchscreen use was associated with decreased overall amount of sleep (beta = −0.146, SE = 0.049, p = 0.003). The unstandardized beta value (−0.26) means that every additional hour of touchscreen use is associated with an overall reduction in sleep of 15.6 minutes.

## Discussion

Data from 715 UK infants and toddlers aged 6–36 months indicated a significant association between the frequency of touchscreen use and sleep quantity (reduced total duration, with reduced duration of night-time and increased daytime sleep), and longer sleep onset (time taken to fall asleep). Every additional hour of tablet use was associated with 15.6 minutes less total sleep (on average, 26.4 minutes less of night-time sleep and 10.8 minutes more of daytime sleep). However, we found no association between touchscreen use and the number of night awakenings. To our knowledge, this is the first study to investigate the association between touchscreen use and infant/toddler sleep. Our results are consistent with a recent meta-review in older children and adolescents illustrating the negative effects of touchscreen use on sleep quality and quantity^27^, extending the findings to younger children under the age of 3. Our results also extend existing reports on the negative effects of TV exposure on sleep in this age group^20^,^21^,^22^,^23^,^24^. We show that, independent of other known factors related to sleep and touchscreen use (age, sex, maternal education and TV exposure), touchscreen use was robustly associated with many sleep attributes.

Studies that have examined media use and sleep have hypothesized four potential mechanisms by which screen time can affect sleep in older children and adolescents. Firstly, electronic media may directly displace the time that children have available for sleep, leading to later bedtime and shorter night-time sleep duration^6^,^7^. Secondly, the content of the media may elevate psychological and physiological arousal, making it more difficult for children to fall asleep and reducing the quality of sleep^28^. Third, the bright blue light from screens can affect the circadian timing through melatonin suppression^29^,^30^, indirectly affecting arousal levels. Fourth, certain heritable traits in a child such as sensation seeking or hyperactivity, which correlate highly with his/her family environment, may also lead to both irregular sleep patterns and increased tablet use. As infants and toddlers have less control over their bedtime schedule, the displacement account is less likely to explain shorter sleep duration than in older children or adolescents, unless parents are, themselves inconsistent and irregular in their night-time routine. However, the portability of touchscreens does allow more flexibility in terms of where such devices are used. As such, some young children who have a touchscreen device in their bedroom may delay falling asleep in favor of playing on a touchscreen or even seek the device when restless in the night. Our finding of increased sleep latency could be due to increased physiological arousal from the media content or from the bright light. However, in the current study we are unable to confirm these hypotheses, as information on the time or nature of exposure is not available. Future research is needed to clarify this relationship by carefully documenting the time and content of use, and, if possible, also measure melatonin levels, physiological arousal and specific temperament traits.

In addition to shorter night-time sleep, increased touchscreen use was also associated with increased daytime sleep. Similarly, increased background TV exposure (not necessarily TV that the child is watching) was also independently associated with reduced daytime sleep. Recent studies in toddlers suggest that daytime nap duration is negatively correlated with night-time sleep duration and sleep onset^31^. As such, touchscreen use may indirectly influence daytime sleep duration by reducing night-time sleep quality, or vice versa. This hypothesis would also be consistent with studies in older children, which found increased media use affecting daytime functioning due to indirect effects of poor quality and quantity of night-time sleep^32^. However, unlike school children, infants and toddlers regularly nap in the day. Thus, the indirect impact of media use on emotional and cognitive functioning through sleep might be lessened in this younger age group, as they are able to ‘catch up’ with their sleep during the day. Our findings indicate that this may not be the case, as despite on average sleeping more during the day, infants and toddlers who spend more time on a touchscreen still spend less overall time sleeping. Future studies that examine the impact of media use on developmental outcomes in this younger age group should take into account both daytime and night-time sleep duration. In addition to the quantity of sleep, Nakagawa and colleagues also reported an association between day time naps that occurred during late afternoon and shorter night-time sleep duration and late sleep onset time^31^. The extent to which increased touchscreen use affects timing of daytime naps cannot be determined in the present study, but warrants further investigation.

Sleep fragmentation, as measured by the number of night awakenings, was not associated with touchscreen use in the current study, when controlling for the known confounds. Previous studies in older children and adolescents that included this variable have reported mixed results: while one study found a positive association between media use and self-report night-time awakenings in adolescents^33^, another study in younger children aged between 4 and 10 years did not^34^. However, sleep fragmentation in the previous and current study was based on self or parent-report, which may not be reliable. A recent study in infants using both parent-report and an objective actigraphy measure of sleep revealed a weak association between the two measures for the frequency of night awakening (r = 0.10), but a strong correlation for nocturnal sleep duration (r = 0.43)^35^. Furthermore, the objective measure of sleep fragmentation was also found to have the strongest impact on infant cognitive development^35^. Although we did not find an association between touchscreen use and sleep quality, this could be due to an underestimation of night awakenings by parent report. It is important that future studies also include an objective measure of sleep fragmentation, either using actigraphy or EEG measures.

In this study, we included a range of specific sleep outcomes encompassing quality, quantity and onset of sleep. Our findings extend the limited knowledge at present on media use and sleep in toddlers beyond TV exposure. Yet, a few limitations should be noted. Firstly, our findings are based on cross-sectional data, therefore a directional relationship between touchscreen use and sleep cannot be established. It is possible that infants and toddlers who use touchscreen devices more frequently also require less sleep. Future longitudinal or intervention studies will be needed to examine the direction of causality. Secondly, as mentioned above, we did not include specific records of the timing, content and location of use. Such information would be crucial in future studies to elucidate the mechanisms by which touchscreen usage impacts sleep patterns. Previous studies in older children do suggest, however, that it is not the exposure to a media device per se that impacts sleep, but the modifiable aspects of media such as content, timing and environment that may have a damaging effect on sleep^24^. Third, the current study only investigated the association between touchscreen use and sleep, it will be important for future studies to establish whether this reduced sleep indirectly impacts cognitive functioning. It is worth noting that touchscreen use may also have positive effect on some aspects of development. In our recent study of the same sample of infants and toddlers, increased active touchscreen use was associated with earlier achievement in fine motor milestones^12^. Thus, total restriction of touchscreen use may limit young children in terms of the potential benefits of these devices. Together, our findings emphasize the need for a more in-depth understanding of how to maximize benefits and minimize negative consequences of this modern technology.

## Methods

### Participants

In total, 715 UK-based parents of 6- to 36-month-old children completed an online questionnaire asking questions about demographic information, their child’s media usage and retrospectively reported developmental milestones. The questionnaire was administered between June 2015 and March 2016. Parents were informed about the broad aim of our online survey: to examine how use of touchscreen devices such as smart phones or tablets might influence infants’ development. Specifically, we were interested in the impact on developmental milestones, sleep and temperament. We encouraged all kinds of users, including babies that had never used a touchscreen. Parents completed the questionnaires, on a voluntary basis, which took on average 15 minutes. The final sample size for each variable varied due to missing data for certain questionnaire elements (see Table 1). An online survey was chosen in order to allow maximum response from a range of socioeconomic groups, varying degrees of touchscreen use and to reach families across the UK. Parents were recruited via the Birkbeck Babylab database, Goldsmiths’ Babylab database and study advertisements from various news agencies, magazines and agencies including National Childbirth Trust (NCT). All methods in the study were carried out in accordance with the latest version of the Declaration of Helsinki, and all experimental protocol were approved by the Birkbeck Psychological Sciences’ ethics board (approval number 141570). Informed consent of the participants was obtained after the nature of the procedures had been fully explained.

Information was collected about the child’s age (mean age = 19.52 months, SD = 8.26 months) and sex (336 females), as well as mother’s educational level (a proxy for family socioeconomic status, SES; “What is the highest degree or level of education the mother of the child has completed?” Responses were “Not applicable”, N = 3; “School leaving qualification”, N = 20; “College”, N = 79; “University”, N = 294; and “Post-graduate”, N = 319). Parents were also asked about child illnesses, but no sleep-related disorders were reported.

### Measures

#### Touchscreen usage and TV exposure

Media questions were derived from existing questionnaires investigating touchscreen usage^8^,^36^,^37^. Parents reported on the frequency of child’s daily touchscreen use: ‘On a typical day, how long does your child spend using a touchscreen device?’. For TV exposure, parents were asked ‘On a typical day, how long is a TV switched on in your home?’.

### Sleep variables

The Brief Screening Questionnaire for Infant Sleep Problems (BISQ^38^) was used to assess infant sleep. This measure has been validated against actigraphy and daily logs, and is sensitive to developmental changes in infant and toddler sleep in the first 3 years^38^. Five sleep variables were obtained by asking parents to report on their child’s (1) night-time sleep duration: ‘How much their child spent sleeping during the night (between 7 pm and 7 am)’; (2) daytime sleep duration: ‘How much does your child spend sleeping during the day (between 7 am and 7 pm)’; 3) number of night awakenings: ‘What is the average number of times your child wakes up per night’; and 4) sleep onset: ‘How long does it take to put your child to sleep in the evening?’.

### Statistical Analysis

Data were initially cleaned using scripts in SPSS^39^ to remove any impossible values due to entry errors. One child’s reported daily touchscreen usage time was removed (recoded as missing) from the current analyses (a clear outlier of 1200 minutes per day which was >19 SDs above the mean). For nocturnal sleep duration, one outlier was removed (18 hours, 6.5 SD above the mean). In addition, scores were capped at 12 hours in line with the questionnaire (duration of sleep at night ‘between 7 pm and 7 am’) with all values above this trimmed back to 12 hours (n = 12). For daytime sleep duration, three responses were removed as they were clear outliers (10–14 hours, 6.5 SDs above the mean). For sleep onset, four responses were > 8 SDs above the mean (10–20 hours), and were removed. No exclusions were made for the average number of night awakenings. Sleep onset and average number of night awakenings were positively skewed and contained zero values. An integer of one was added to all values in these variables, which were then transformed to normal using the Zero-skewness Box-Cox transformation (bcskew0 command in Stata^40^). Duration of night-time sleep was slightly skewed (skewness of −1.25), but rather than transform this variable, we accounted for this slight non-normality by applying an estimator with robust standard errors in the analysis. To test the relationship between touchscreen usage and sleep, a saturated path analysis model was estimated in Mplus^41^, using maximum likelihood with robust standard errors (MLR), covarying for age, sex, maternal education and background TV exposure. The MLR estimator assumes data are missing at random^42^, where missingness relates only to observed variables. Standardized model results (STDYX) are presented.

## Additional Information

**How to cite this article**: Cheung, C. H. M. *et al*. Daily touchscreen use in infants and toddlers is associated with reduced sleep and delayed sleep onset. *Sci. Rep.* **7**, 46104; doi: 10.1038/srep46104 (2017).

**Publisher's note:** Springer Nature remains neutral with regard to jurisdictional claims in published maps and institutional affiliations.

## Figures and Tables

**Table 1:** Descriptive statistics: Parent reported touchscreen use and sleep patterns in 6- to 36-month-olds.

		Age quartiles				Total
		6–11 m	12–18 m	19–25 m	26–36 m	6–36 m
Age (months)	M (SD) N	8.99 (1.82) 134	14.40 (2.19) 215	21.94 (2.07) 179	30.64 (3.07) 187	19.52 (8.26) 715
Male	% N	51.49 134	53.02 215	55.87 179	51.34 187	53.01 715
Background TV (minutes)	M (SD) N	209.72 (186.08) 123	189.62 (172.70) 194	187.00 (162.99) 145	219.01 (183.24) 151	200.27 (175.99) 613
Touchscreen use (minutes)	M (SD) N	8.53 (15.54) 123	18.80 (36.83) 194	25.18 (37.46) 145	44.11 (47.75) 150	24.45 (38.98) 612
Night-time sleep duration (minutes)	M (SD) N	637.24 (64.81) 116	647.24 (50.13) 176	651.25 (60.12) 132	643.21 (61.81) 130	645.16 58.66 554
Daytime sleep duration (minutes)	M (SD) N	139.05 (47.27) 116	122.47 (38.40) 176	100.57 (40.28) 132	68.25 (53.20) 126	108.29 (51.15) 552
Total sleep (sum of night-time & daytime sleep)	M (SD) N	776.29 (64.94) 116	769.31 (51.84) 175	751.82 (71.09) 132	711.63 (68.23) 126	753.34 (67.84) 549
Average number of night awakenings	M (SD) N	2.00 (1.73) 116	1.29 (1.33) 177	0.91 (1.01) 133	0.58 (0.81) 130	1.17 (1.35) 556
Sleep onset	M (SD) N	22.80 (23.69) 116	21.54 (16.53) 177	22.22 (16.97) 131	29.34 (27.86) 130	23.79 (21.49) 551

**Table 2:** Correlations of the sleep variables.

	Night-time sleep duration	Daytime sleep duration	Sleep onset
Daytime sleep duration	−0.24^^^	—	—
Sleep onset	−0.32*	−0.02	—
Average number of awakenings	−0.31*	0.11*	0.19*

Pearson’s correlations were conducted for the correlation between night-time and daytime duration as these two variables were normally distributed. Spearman’s rho correlations were conducted for all remaining comparisons as sleep onset and average number of awakenings were not normally distributed.

^^^Pearson’s r correlations, p < 0.01.

*Spearman’s rho correlations, p < 0.01.
